# Larvicidal Effects and Phytochemical Analysis of Myrrh, *Commiphora myrrh* Chloroform, Methanol, and Acetone Extracts Against Dengue Vector *Aedes aegypti* L. (Diptera: Culicidae)

**DOI:** 10.3390/ijms26168050

**Published:** 2025-08-20

**Authors:** Abadi M. Mashlawi, Hanan Bosly, Amal Naif Alshammari, Naimah Asid H. Alanazi, Mohammed A. Akeel, Amani Alhejely, Fahdah Ayed Alshammari, Mohammed Abdullah Jeraiby, Naser Ahmed Alkenani, Salama A. Salama

**Affiliations:** 1 Department of Biology, College of Science, Jazan University, Jazan 45142, Saudi Arabia; amashlawi@jazanu.edu.sa (A.M.M.); habosly@jazanu.edu.sa (H.B.); 2 Environment and Nature Research Center, Jazan University, Jazan 45142, Saudi Arabia; 3 Biology Department, Darb University College, Jazan University, Jazan 45142, Saudi Arabia; ashammari@jazanu.edu.sa (A.N.A.); alhejely@jazanu.edu.sa (A.A.); 4 Department of Biology, College of Sciences, University of Ha’il, Ha’il 53962, Saudi Arabia; n.alenezy@uoh.edu.sa; 5 Human Anatomy Department, Faculty of Medicine, Jazan University, Jazan 45142, Saudi Arabia; m.akeel@jazanu.edu.sa; 6 Department of Biology, College of Science, Northern Border University, Arar 73241, Saudi Arabia; fahdah.ayed@nbu.edu.sa; 7 Department of Basic Medical Science, College of Medicine, Jazan University, Jazan 45142, Saudi Arabia; mmojer@jazanu.edu.sa; 8 Department of Biological Sciences, Faculty of Science, King Abdulaziz University, Jeddah 21589, Saudi Arabia; nalkenani@kau.edu.sa; 9 Zoology Department, Faculty of Science, Damanhur University, Damanhur 22511, Egypt

**Keywords:** *Commiphora myrrha*, *Aedes aegypti*, resin, larvicidal activity, GC/MS analysis, mosquitoes

## Abstract

Mosquitoes pose a significant problem worldwide because of the diseases they transmit. Due to its antimicrobial and disinfectant properties, *Commiphora myrrha* (*C. myrrha*) has long been a popular choice in traditional medicine. This study aimed to extract *C. myrrha* using three different solvents—methanol, acetone, and chloroform—to identify their biochemical components and assess their larvicidal activity. The extracts were analyzed using gas chromatography–mass spectrometry, and their effects were evaluated against *Aedes aegypti*. We identified 29, 41, and 19 phytoconstituents in the acetone, methanol, and chloroform extracts, respectively, with most belonging to the sesquiterpene and phenol categories. Larval mortality rates were recorded as follows: chloroform (100%), methanol (90%), and acetone (95%) extracts of *C. myrrha* at a concentration of 1000 ppm, 24 h post-treatment. After 72 h, the *C. myrrha* extracts showed effectiveness with LC_50_ values of 118.33, 127.67, and 142.13 ppm for chloroform, acetone, and methanol, respectively. The chloroform extract was the most effective in reducing the average number of eggs laid per day (234 eggs) compared to the untreated control group (1513 eggs) at 1000 ppm. These findings provide scientific evidence of the larvicidal efficacy of *C. myrrha* extracts and serve as valuable resources for developing plant-based pharmaceuticals.

## 1. Introduction

Diseases transmitted by mosquitoes pose significant threats not only to human health and domesticated animals but also to economic and social development. This impact is largely due to their widespread distribution across diverse environments, the rapid emergence of resistance among mosquito vectors, and the absence of effective vaccines [1,2,3]. Dengue fever has emerged as the most prevalent virus transmitted by mosquitoes in the modern era, posing serious economic and public health concerns [4,5]. It affects every tropical and subtropical region of the globe, posing serious economic and public health concerns [4,5]. Dengue fever has increased by 30 times in the last five decades, and the disease currently affects up to 390 million people each year [6]. The historically high infection rate of the disease has significantly hindered growth and economic progress, primarily due to lost production and the expenses associated with mosquito control [7].

Mosquitoes and other pests that threaten public health are often controlled using synthetic chemical pesticides, such as pyrethroids and organophosphates [8]. However, the widespread use of these chemicals has led to two significant issues: first, mosquitoes have developed resistance to many pesticides [9]; second, these chemicals can cause considerable harm to humans [10,11]. Consequently, the development of safe and environmentally friendly insecticides is essential for public health. Most known organic chemicals are derived from natural sources, with secondary metabolites playing a vital role in the advancement of modern synthetic organic chemistry. For instance, essential oils are plant-based insecticides that typically have minimal or no impact on non-target organisms and are accessible in many regions facing mosquito-borne diseases [12,13]. Therefore, these chemical compounds could be further explored for the development of natural-source medications or even bioinsecticides that pose less risk to mammals and the environment [14].

*Commiphora* is a member of the Burseraceae family, primarily found in Saudi Arabia but also present across Asia and northern Africa [15]. These plants have a long history of use in traditional medicine, largely due to their antibacterial, antiseptic, and analgesic properties [15,16]. Muturi et al. found that mosquito larvicides could potentially be derived from the essential oil of *Commiphora erythraea* [17]. *Commiphora myrrha* (*C. myrrha*) is a well-known herbal remedy utilized for various health conditions [18]. The aromatic oleo-gum resin obtained from *C. myrrha* is recognized for its efficacy as an antibacterial agent and offers a range of beneficial applications. These include treating conditions such as brucellosis, glandular fever, sinusitis, gingivitis, mouth ulcers, and parasitic infections [18]. The volatile oils and crude extracts of *C. myrrha* exhibit a wide array of biological activities, including cytotoxicity, anesthesia, and anti-inflammatory, antiviral, and antibacterial effects [18,19,20].

To safeguard public health, it is essential to discover environmentally responsible methods for controlling microbes and pests. This study aims to identify the bioactive components and allelochemicals found in crude extracts of medicinal plant resins, specifically those from *C. myrrha*. Previous research has largely concentrated on assessing the phytoconstituents and biological activities of resins derived from various *Commiphora* species. However, there is a notable lack of detailed reports concerning the phytoconstituents of *C. myrrha* extracted using different solvents with varying polarities. Additionally, there is insufficient comparative analysis of their biological activities, particularly in relation to larvicidal properties. Therefore, this study seeks to investigate the phytochemical constituents of *C. myrrha* extracted with solvents of varying polarities and to evaluate their larvicidal properties. The extraction of *C. myrrha* was carried out using three solvents: methanol, acetone, and chloroform. Each plant extract was analyzed separately to identify its chemical constituents and assess its biological properties. This analysis utilized gas chromatography–mass spectrometry, and the resulting phytoconstituents were tested for their effectiveness in controlling *Aedes aegypti* (*A. aegypti*) larvae.

## 2. Results

### 2.1. Biochemical Analysis

**Figure 1:**
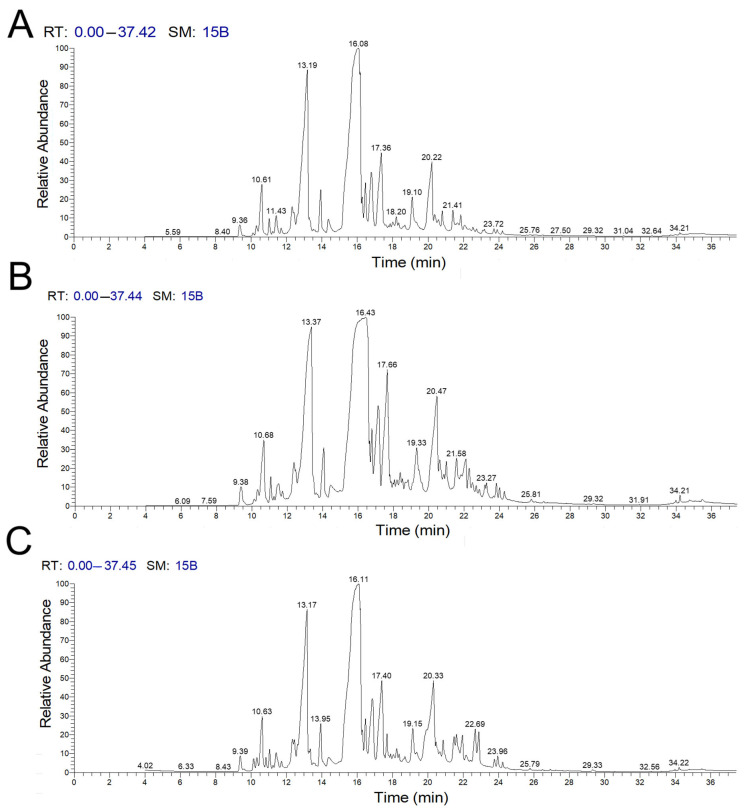
The chromatogram shows various components identified from the (**A**) acetone, (**B**) methanol, and (**C**) chloroform extracts of *C. myrrha*, obtained through GC-MS analysis.

Figure 1 presents a chromatogram that illustrates the relationship between the retention times of various components and their relative quantities in the extracted plant material. The analysis of the chemical components in *C. myrrha* extracts indicates that majority of the identified compounds were sesquiterpenes and phenols (see Table 1, Table 2 and Table 3). Among the 29 compounds identified in the acetone extract of *C. myrrha*, the most abundant was naphthalene 4-methoxy-1,2,6,8-tetramethyl. In the methanol extract, a total of 41 compounds were identified, with trans-benzofuran 6-ethenyl-4,5,6,7-tetrahydro-3,6-dimethyl-5-isopropenyl being the most prevalent. In the chloroform extract of *C. myrrha*, 29 compounds were identified, with (4aS,8aS)-3,8a-Dimethyl-5-methylene-4,4a,5,6,8a,9-hexahydronaphtho[2,3-b]furan being the most abundant.

### 2.2. Effect of the C. myrrha Resin Extracts on Larvae

The results in Table 4 indicate that there was no larval mortality in any of the control untreated groups. In contrast, all plant extracts demonstrated larvicidal activity, with the chloroform extract achieving the highest (ANOVA, *p*-value < 0.0001) larval mortality at 100% efficacy. This was followed by the acetone extract at 95% and the methanol extract at 90%, with all extracts tested at a concentration of 1000 ppm, 24 h after treatment. Furthermore, the larval mortality rate was significantly (ANOVA, *p*-value < 0.0001) higher in the chloroform extract compared to the acetone and methanol extracts, also at 1000 ppm, 24 h post-treatment.

Furthermore, Table 4 shows that the percentage of pupal mortality was significantly (ANOVA, *p*-value < 0.0001) higher in the plant resin extracts compared to the untreated control groups, except for the chloroform extract. Specifically, pupal mortality increased by 99.41% and 50.15% for the acetone and methanol extracts, respectively, at a concentration of 1000 ppm, 24 h post-treatment. However, there was no significant (ANOVA, *p*-value > 0.05) difference in pupal mortality between the untreated control groups and the chloroform extract at 1000 ppm, 24 h post-treatment.

It is also noted in Table 4 that the percentage of adult emergence was higher in the untreated control groups compared to the plant resin extracts. In contrast, the adult emergence significantly (ANOVA, *p*-value < 0.0001) decreased by 98.30%, 94.83%, and 98.33% for the acetone, methanol, and chloroform extracts, respectively, when applied at 1000 ppm, 24 h post-treatment, in comparison to the untreated control groups. Among all the plant extracts tested, the chloroform extract demonstrated the highest effectiveness in reducing the adult emergence percentage.

The larvicidal effects of the plant resin *C. myrrha* on *A. aegypti*, as shown in Table 5, Table 6 and Table 7, are influenced by both the type of solvent used for extraction and the concentration of the extracts. In all control untreated groups, there was no larval mortality after 24, 48, and 72 h. When the concentration increased from 100 ppm to 1000 ppm, the mortality rate significantly (ANOVA, *p*-value < 0.0001) increased by factors of 5.33, 6.71, and 4.99 for acetone, methanol, and chloroform extracts, respectively, 24 h after treatment. Similarly, at 48 h post-treatment, the mortality rate rose (ANOVA, *p*-value < 0.0001) by factors of 3.0, 3.83, and 2.53 for acetone, methanol, and chloroform extracts, respectively, with the concentration raised from 100 ppm to 1000 ppm. At 72 h post-treatment, the mortality rate increased (ANOVA, *p*-value < 0.0001) by factors of 1.61, 2.0, and 1.5 for acetone, methanol, and chloroform extracts, respectively, following the same increase in concentration.

Furthermore, Table 5, Table 6 and Table 7 illustrate that the larvicidal effects of *C. myrrha* on *A. aegypti* were enhanced with longer exposure times to the plant resin extracts. The mortality percentage of the larvae significantly (ANOVA, *p*-value < 0.0001) increased by 22.22% and 33.33% for the acetone extract at 500 ppm when the exposure time was extended from 24 h to 72 h post-treatment. Similarly, for the methanol extract at 500 ppm, the mortality percentage exhibited significant (ANOVA, *p*-value < 0.0001) increases of 18.19% and 36.37% with the same change in exposure time. Furthermore, the mortality percentage for the acetone extract at 500 ppm increased (ANOVA, *p*-value < 0.0001) by 20.83% and 20% when the exposure time was lengthened from 24 h to 72 h post-treatment.

It is also noted in Table 5, Table 6 and Table 7 that the chloroform extract exhibited the highest (ANOVA, *p*-value < 0.0001) percentage of larval mortality among all plant extracts at 24 h post-treatment; however, it showed no significant (ANOVA, *p*-value > 0.05) differences at 72 h after treatment.

The LC50 values for the chloroform extract significantly (ANOVA, *p*-value < 0.0001) decreased 24 h after treatment, showing reductions of 8.91% compared to acetone and 20.39% compared to methanol. Additionally, 48 h post-treatment, the LC50 values for the chloroform extract decreased significantly (ANOVA, *p*-value < 0.0001) by 9.52% compared to acetone and by 21.65% compared to methanol. Furthermore, 72 h after treatment, the LC50 values for the chloroform extract decreased significantly (ANOVA, *p*-value < 0.0001) by 7.31% compared to acetone and by 16.75% compared to methanol.

The rate of deposited eggs (Table 8) varied significantly based on the plant resin extracts of *C. myrrha*. The chloroform extract was particularly effective in reducing the daily average number of eggs laid, followed by the acetone and methanol extracts at a concentration of 1000 ppm when compared to untreated control groups. The chloroform extract led to a substantial (ANOVA, *p*-value < 0.0001) decrease in the rate of deposited eggs by 38.75% compared to acetone and by 44.81% compared to methanol, both at 1000 ppm.

Furthermore, Table 8 indicates that the hatching rate was significantly (ANOVA, *p*-value < 0.0001) lower in the chloroform extract, followed by acetone and methanol when compared to untreated control groups at a concentration of 1000 ppm. The hatching rate for the chloroform extract decreased significantly (ANOVA, *p*-value < 0.0001) by 74.22% and 81.58% compared to the acetone and methanol extracts at 1000 ppm, respectively.

It also noted in Table 8 that fecundity was significantly (ANOVA, *p*-value < 0.0001) lower in the chloroform extract, followed by acetone and methanol, at a concentration of 1000 ppm compared to the untreated control groups. The fecundity decreased significantly (ANOVA, *p*-value < 0.0001) for the chloroform extract by 42.81% and 46.95% when compared to acetone and methanol, respectively, at 1000 ppm.

The chloroform extract of *C. myrrha* resin was the most effective ANOVA, *p*-value < 0.0001) in causing fatality in egg embryos, followed by acetone and methanol when compared to untreated control groups. The fatality rate for the chloroform extract increased significantly by 76.09% compared to acetone and by 244.39% compared to methanol at a concentration of 1000 ppm.

It is evident that there is an inverse correlation between plant resin extracts and non-hatching rates when compared to untreated groups. Statistical analysis using ANOVA indicated that the *C. myrrha* extracts significantly impacted the reduction in female eggs (F = 18.78, df = 4, *p* = 0.001).

## 3. Discussion

It remains challenging to find effective alternatives to conventional insecticides in the battle against mosquitoes. In this context, there is increasing interest in exploring natural products for their potential insecticidal properties [16]. The medicinal plant *C. myrrha* is particularly recognized for its oleo-gum resin. Beyond their numerous pharmacological applications, the resins obtained from this species have demonstrated potential in treating inflammatory diseases, oral ulcers, pain, fractures, gastrointestinal disorders, microbial infections, and wounds [16,21,22,23]. In Unani medicine, gums serve various functions, including being astringent, antiseptic, carminative, emmenagogue, expectorant, and stomachic. They are also employed externally for wound treatment, the prevention of epidemic diseases, and alleviating gout and joint pain [16].

The extraction method and the choice of solvents significantly influence the quantity and types of secondary metabolites present in medicinal plants. Different solvents, based on their polarity, are known to yield distinct phytomolecules during the preparation of plant extracts [24]. Numerous studies have demonstrated that solvents can alter the diversity and biological activity of secondary metabolites [25]. Therefore, selecting appropriate extraction solvents and methods is essential for enhancing the biological properties of phytoconstituents. Utilizing various solvents to obtain the same plant extract facilitates beneficial comparative biological studies. Without a clear understanding of how solvents affect our *C. myrrha* extracts, achieving the desired biological effects may not be possible. In this investigation, three polarity-based solvents—methanol, acetone, and chloroform—were employed to extract *C. myrrha*.

Our results from the GC-MS analysis indicated that the most abundant compounds in the *C. myrrha* extracts included sesquiterpenes, phenols, sesquiterpene lactones, and aldehydes. The chemical components of *C. myrrha* were compared in this study to those reported in earlier research [26,27,28,29]. Ahamad et al. identified several organic and inorganic components in the *C. myrrha* ethanolic extract. Among the 27 organic compounds they estimated, key components included limonene, curzerene, germacrene B, isocericenine, myrcenol, beta-selinene, and spathulenol [26]. The primary components identified by Ammar et al. in the chloroform extract of the oleo-gum-resin of *C. myrrha* were Z-[gamma]-bisabolene and [gamma]-elemene [27]. Alabdalall et al. identified the principal components of *C. myrrha* as furanoeudesma-1,3-diene, curzerene, β-elemene, and various forms of germacrene (B, D, and A) [28]. According to Baz et al., the main chemical components found in *C. myrrha* included 4,4′-Dimethyl-2,2′-dimethylenebicyclohexyl-3,3′-diene and α-pinene [29]. Several factors, such as solvent selection, environmental and climatic conditions, and geographic influences, may account for the observed differences in the chemical compositions of *C. myrrha*.

Findings from this study indicate that extracts from *C. myrrha* are more effective than untreated control groups in reducing *A. aegypti* larvae populations. At 24, 48, and 72 h post-treatment, our data demonstrated that chloroform, at a concentration of 1000 ppm, was the most effective solvent. Acetone and methanol extracts ranked second and third, respectively. The fecundity of *Aedes aegypti* larvae was significantly altered, as extracts of *C. myrrha* exhibited various biological effects, including reduced egg deposition and hatching rates. The efficacy of *C. myrrha* is attributed to many secondary metabolites, including phenols, aromatic terpenoids, terpenes, sesquiterpenes, eugenol, ketones, fatty alcohols, and cumin aldehyde, which are present in *C. myrrha* resin extracts [20].

In their study on mosquito larvae, Baz et al. found that acetone extracts of *C. myrrha* at 1500 ppm resulted in the highest mortality rates (LC50 values of 623.52 and 300.63 ppm) among the five plant species tested [29]. In contrast, the current study presents more promising results, with an LC50 ranging from 118.33 to 142.13 ppm, indicating high efficacy. Additionally, the oil-resin derived from *Commiphora* species is well-known for its effectiveness against mosquito larvae [17,30]. Samwel et al. isolated two larvicidal compounds, particularly arabinofuranoside tridecanols, from the exudate of *Commiphora merkeri* [31]. These compounds exhibited larvicidal activity against *A. aegypti*, with LC50 values of 40.66 µg/mL and 33.79 µg/mL, respectively. The results of this study are comparable to those of Samwel et al. [31], although the former focused on pure compounds while the latter examined extracts.

The research highlights certain limitations, particularly the necessity of a comparator drug to function as a positive control. Studies that examine novel extracts as insecticides need a positive control to accurately assess treatment efficacy. Consequently, our upcoming research will primarily focus on investigating the effects of various phytochemicals derived from *C. myrrha* in comparison to positive control insecticides like azadirachtin.

## 4. Materials and Methods

### 4.1. Plants Materials

**Figure 2:**
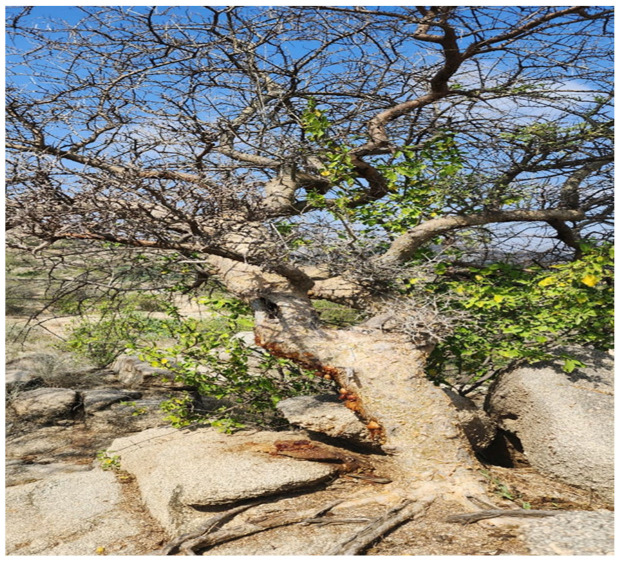
Collection of *C. myrrha* plant resin.

*C. myrrha* gum resin was collected in the summer from a four-year-old tree from various locations in Wadi Lajab, southwest of the Jazan region, Saudi Arabia (Figure 2). During the harvesting process, the tree bark was tapped three times with an ax. The tree was then left exposed to sunlight to allow the resin to dry and harden. Once it had solidified, the resin was carefully scraped off the bark. After harvesting, the resins were collected in a glass basin and purified by removing any remaining impurities, such as bark, leaves, and sand. The Flora and Phytotaxonomic Section in the Biology Department of Jazan University’s Faculty of Science was responsible for identifying the plant resins. The herbarium of Jazan University’s Biology Department holds samples, which have been assigned voucher numbers.

### 4.2. Preparation of Crude Extracts

In a 250 mL beaker, 20 g of resin was weighed and mixed with 100 mL of each solvent to create chloroform, methanol, and acetone extracts of *C. myrrha*. The beakers were placed in a dark environment and sealed to prevent solvent evaporation for 24 h. A magnetic shaker (Heidolph^®^ Unimax Orbital Shaker 1010, Schwabach, Germany) stirred the three solvent beakers at 300 rpm for 5 min at 29 °C. The mixtures were filtered through Whatman No. 4 filters to collect the extracts. Two additional applications of fresh solvent were performed to extract any remaining residues. The extracts were obtained from the combined supernatants of each solvent by evaporating under vacuum (Heidolph™ Instruments Hei-VAP, Heidolph Scientific Products GmbH, Schwabach, Germany) at 40 °C. The resulting crude extracts were used for further research.

### 4.3. Gas Chromatography–Mass Spectrometry (GC-MS) Analysis

To analyze the *C. myrrha* resin extract using gas chromatography–mass spectrometry (GC-MS, Thermo Scientific, located in Austin, TX, USA), a direct capillary column TG-5MS (30 m × 0.25 mm × 0.25 µm film thickness) was utilized [32]. The column oven temperature was initially set at 50 °C and then increased to 250 °C at a rate of 5 °C/min. This temperature was maintained for 2 min before being rapidly raised to 300 °C at a rate of 30 °C/min, where it was held for an additional 2 min. At 250 °C, the injector temperature was maintained constant, while the MS transfer line was set to 260 °C. Helium was used as the carrier gas, supplied at a steady rate of 1 mL/min. The Autosampler AS1300 (Thermo-Scientific, Austin, TX, USA) was employed to automatically inject 1 µL of the sample. Electron ionization (EI) mass spectrometry data were collected in packed scan mode with an ionization energy of 70 eV, covering an m/z range of 50–500. The ion source temperature was calibrated to 250 °C.

### 4.4. Identification of Individual Components of Plant Extract

By comparing the retention times and mass spectra of the various components with data from the mass spectrometry databases WILEY 09 and NIST 11, we were able to determine the chemical composition of each extract.

### 4.5. Aedes Aegypti Colony

Jazan University in Saudi Arabia provided the *A. aegypti* L. eggs as part of the Biology Department within the Faculty of Science. To aid hatching, the egg ovitrap was placed in a tray containing food and dechlorinated water, with added yeast. After two days, the first-instar larvae were transferred to a fresh tray of clean water using plastic pipettes. The larvae trays were kept at a temperature of 27 ± 2 °C and a relative humidity of 75 ± 5%. Monitoring was conducted using a ROTRONIC HygroClip probe (HC2A-S, Rotronic, Bassersdorf, Switzerland), and the trays were subjected to a dark–light cycle every 13:11. The *A. aegypti* larvae were fed commercial dog biscuits until they reached the pupal stage of metamorphosis. Subsequently, they were placed in a container with clean water to develop into adult mosquitoes. Once fully grown, the mosquitoes were released into a cage. They were provided with a 10% sucrose solution for feeding.

### 4.6. Larvicidal Bioassay

Methanol, acetone, and chloroform extracts from the *C. myrrha* were evaporated. The plant resin from each beaker was weighed and mixed with 100 mL of distilled water to create a stock solution. From this stock solution, varying concentrations of methanol, acetone, and chloroform plant resin were prepared and tested against *Aedes aegypti* larvae. The concentrations used were 100, 200, 300, 400, 500, and 1000 ppm. The WHO protocol was followed for this experiment [33,34]. For each concentration, twenty larvae in the late third or early fourth instar were selected and placed in a 250 mL glass beaker filled with dechlorinated water. Each test was repeated three times, using a negative control group that did not receive any treatment. Dead pupae and larvae were collected daily, and records of larval mortality were maintained. Following treatment, the larvae’s growth was monitored every day until pupation and adult emergence.

When larvae did not respond to physical stimuli, such as a blunt pointer touched to the cervical region, or when exposed to light, they were deemed dead. Mortality was observed daily until adult emergence, and deceased larvae and pupae were removed from the containers [35]. The percent of mortality was calculated using the following formula [36]:

(1) $$\mathrm{Larval}\;\mathrm{mortality}\;\mathrm{percentage}\;=\;\left[\frac{\mathrm{Number}\;\mathrm{of}\;\mathrm{dead}\;\mathrm{larvae}\;}{\mathrm{number}\;\mathrm{of}\;\mathrm{treated}\;\mathrm{larvae}\;}\right]\;\times \;100$$

We used Abbott’s formula to correct for mortality when pupae were present [37]. Consequently, the following is how the total number of larvae that died in each treatment was determined:

(2) $$\mathrm{Larval}\;\mathrm{mortality}\;\mathrm{percentage}\;=\;\left[\frac{\mathrm{Number}\;\mathrm{of}\;\mathrm{dead}\;\mathrm{larvae}}{\mathrm{Number}\;\mathrm{of}\;\mathrm{exposed}\;\mathrm{larvae}\;-\;\mathrm{Number}\;\mathrm{of}\;\mathrm{pupae}}\right]\;\times \;100$$

Failure to reach the adult stage was a sign of pupal death [38]. The pupal mortality percentage was calculated as follows:

(3) $$\mathrm{Pupal}\;\mathrm{mortality}\;\mathrm{percentage}\;=\;\left[\frac{\mathrm{number}\;\mathrm{of}\;\mathrm{pupae}\;\mathrm{that}\;\mathrm{died}\;}{\mathrm{number}\;\mathrm{of}\;\mathrm{pupae}\;\mathrm{produced}}\right]\;\times \;100$$

(4) $$\mathrm{The}\;\mathrm{adult}\;\mathrm{emergence}\%=\left[\frac{\mathrm{number}\;\mathrm{of}\;\mathrm{adults}\;\mathrm{who}\;\mathrm{have}\;\mathrm{emerged}}{\mathrm{number}\;\mathrm{of}\;\mathrm{pupae}\;\mathrm{produced}}\right]\times 100$$

A breeding cage was utilized to raise mosquito eggs collected from the existing colony. In 100 mL plastic containers, the eggs were exposed to various concentrations of methanol, acetone, and chloroform plant resin (100, 200, 300, 400, 500, and 1000 ppm), along with a control group. After treatment, the eggs or egg rafts from each concentration were placed in separate cups of distilled water. Following this, the eggs were counted using a stereomicroscope and assessed for hatching [39]. The total number of eggs, the number laid by a single female (fecundity), and the percentage of egg hatching were calculated as follows:

(5) $$\mathrm{The}\;\mathrm{egg}\;\mathrm{hatching}\%\;=\;\left[\frac{Number\;of\;Egg\;hatching}{number\;of\;egg\;laid}\right]\;\times \;100$$

Similarly, the following formula was used to obtain the fecundity percentage:

(6) $$\mathrm{The}\;\mathrm{fecundity}\%\;=\;\left[\frac{\mathrm{Number}\;\mathrm{of}\;\mathrm{dead}\;\mathrm{larvae}}{number\;of\;treated\;larvea}\right]\;\times \;100.$$

### 4.7. Data Analysis

Using the computer program PASW Statistics 2009 (SPSS version 22), the biological data were treated to one-way analysis of variance (ANOVA), Duncan’s multiple range tests, and probit analysis for calculating the lethal values.

## 5. Conclusions

This study evaluated the larvicidal effects of three resin extracts from *C. myrrha*—acetone, methanol, and chloroform—to determine how the choice of solvent for extraction influenced the secondary metabolites present in the plants. The chemical compositions of the extracts varied significantly, and the quantities of several important phytoconstituents also differed. Sesquiterpenes and phenols were identified as the most prevalent classes of phytoconstituents in *C. myrrha* extracts. The larvicidal properties were most pronounced in the chloroform extract of *C. myrrha*. These findings support the traditional use of chloroform extract from *C. myrrha* in medicinal applications. Additionally, this extract could provide valuable insights for those interested in developing plant-derived medicines. In conclusion, this research adds to the growing body of literature that explores natural alternatives to synthetic pesticides, with a particular focus on *C. myrrha* resin.

## Figures and Tables

**Table 1:** The primary chemical constituents found in acetone extracts of *C. myrrha*.

No.	RT	Name of the Compound	M. F	M. Wt	Area (%)
1	9.35	Cyclohexene,4-ethenyl-4-methyl-3-(1-methylethenyl)-1-(1-methylethyl)-, (3R-trans)-	C15H24	204	0.92
2	10.62	Cyclohexane,1-ethenyl-1-methyl-2,4-bis(1-methylethenyl)-, [1S-(1à,2á,4á)]-	C15H24	204	4.06
3	11.03	Caryophyllene	C15H24	204	0.95
4	11.43	Ç-Elemene	C15H24	204	2.57
5	12.60	À-selinene	C15H24	204	0.87
6	13.20	Curzerene	C15H20O	216	11.68
7	13.35	Naphthalene,1,2,3,5,6,8a-hexahydro-4,7-dimethyl-1-(1-methylethyl)-, (1S-cis)-	C15H24	204	0.19
8	13.95	1,5-Cyclodecadiene, 1,5-dimethyl-8-(1-methylethylidene)-, (E,E)-	C15H24	204	3.09
9	16.10	Naphthalene, 4-methoxy-1,2,6,8-tetramethyl-	C15H18O	214	32.23
10	16.17	(4as,8as)-3,8a-Dimethyl-5-methylene-4,4a,5,6,8a,9-hexahydronaphtho[2,3-b]furan	C15H18O	214	6.86
11	16.27	5-isopropenyl-3,6-dimethyl-6-vinyl-4,5,6,7-tetrahydro-1-benzofuran	C15H20O	216	0.60
12	16.48	Cyclohexanemethanol,4-ethenyl-à,à,4-trimethyl-3-(1-methylethenyl)-, acetate, [1R-(1à,3à,4á)]-	C17H28O2	264	2.32
13	17.36	(R,5E,9E)-8-Methoxy-3,6,10-trimethyl-4,7,8,11-tetrahydrocyclodeca[b]furan	C16H22O2	246	15.19
14	17.85	7-tetracyclo[6.2.1.0(3.8)0(3.9)]undecanol,4,4,11,11-tetramethyl-	C15H24O	220	0.15
15	18.01	Azulene,1,2,3,4,5,6,7,8-octahydro-1,4-dimethyl-7-(1-methylethylidene)-, (1s-cis)-	C15H24	204	0.95
16	18.60	Reynosin	C15H20O3	248	0.13
17	18.68	6-(1-hydroxymethyl-vinyl)-4,8a-dimethyl-3,5,6,7,8,8a-hexahydro-1h-naphthalen-2-one	C15H22O2	234	0.26
18	19.11	Acetic acid, 6-(1-hydroxymethyl-vinyl)-4,8a-dimethyl-3-oxo-1,2,3,5,6,7,8,8a-octahydronaphthalen-2-yl ester	C17H24O4	292	1.98
19	19.35	Cholan-24-oic Acid, 3,7,12-trihydroxy-(3à,5á,7à,12à)-	C24H40O5	408	2.25
20	20.23	(4as,7r,8as)-6,9,9-trimethyl-4,4a,7,8,8a,9-hexahydronaphtho[2,3-b]furan-7-ol	C15H20O2	323	8.98
21	21.41	4a-Methyl-1-methylene-1,2,3,4,4a,9,10,10a-octahydrophenanthrene	C16H20	212	1.03
22	21.66	17Alpha-ethynyl-17beta-hydroxy-6beta-methoxy-3alpha,5-cyclo-5alpha-androstan-19-oic acid	C22H30O4	358	0.14
23	21.85	Furosardonin A	C15H20O2	232	0.71
24	22.06	Gazaniolide	C15H18O2	230	0.56
25	22.73	Ethanone, 2-(2,2,4,6,6-pentamethylcyclohexylidene)-1-phenyl-	C19H26O	270	0.19
26	23.18	10-Octadecenoic acid, methyl ester	C19H36O2	296	0.44
27	23.72	1,3,6,10-cyclotetradecatetraene, 3,7,11-trimethyl-14-(1-methylethyl)-, [s-(e,z,e,e)]-	C20H32	272	0.28
28	23.90	Nerolidol-epoxyacetate	C17H28O4	296	0.26
29	34.21	24-Norursa-3,12-diene	C29H46	394	0.19

**Table 2:** The primary chemical constituents found in methanol extracts of *C. myrrha*.

No.	RT	Name of the Compound	M. F	M. Wt	Area (%)
1	9.36	Cyclohexene, 4-ethenyl-4-methyl-3-(1-methylethenyl)-1-(1-methylethyl)-, (3R-trans)-	C15H24	204	1.12
2	10.12	Copaene	C15H24	204	0.18
3	10.68	Cyclohexane, 1-ethenyl-1-methyl-2,4-bis(1-methylethenyl)-, [1S-(1à,2á,4á)]-	C15H24	204	5.71
5	12.39	1,6-cyclodecadiene, 1-methyl-5-methylene-8-(1-methylethyl)-, [S-(E,E)]-	C15H24	204	2.85
6	13.38	Benzofuran, 6-ethenyl-4,5,6,7-tetrahydro-3,6-dimethyl-5-isopropenyl-, trans-	C15H20O	216	24.5
7	13.64	Aromandendrene	C15H24	104	0.11
8	14.07	1,5-Cyclodecadiene, 1,5-dimethyl-8-(1-methylethylidene)-, (E,E)-	C15H24	204	3.31
9	14.44	3,5,8a-Trimethyl-4,6,8a,9-tetrahydronaphtho[2,3-b]furan	C15H18O	214	1.77
10	16.51	Naphthalene, 4-methoxy-1,2,6,8-tetramethyl-	C15H18O	214	15.02
11	16.57	(4aS,8aS)-3,8a-Dimethyl-5-methylene-4,4a,5,6,8a,9-hexahydronaphtho[2,3-b]furan	C15H18O	214	3.08
12	16.80	Cyclohexanemethanol, 4-ethenyl-à,à,4-trimethyl-3-(1-methylethenyl)-, acetate, [1R-(1à,3à,4á)]-	C17H28O2	264	1.99
13	17.14	(5E)-3,6,10-trimethyl-4,7,8,11-tetrahydrocyclodeca[b]furan	C15H20O	216	6.58
14	17.67	(R,5E,9E)-8-Methoxy-3,6,10-trimethyl-4,7,8,11-tetrahydrocyclodeca[b]furan	C16H22O2	246	5.62
15	17.73	3-ethyl-2,6-naphthlenediol	C12H12O2	188	1.20
16	17.80	Santamarine	C15H20O3	248	0.17
18	18.51	á-Guaiene	C15H24	204	0.74
19	18.67	Cholan-24-oic acid, 3,7,12-trihydroxy-, (3à,5á,7à,12à)-	C24H40O5	408	0.38
20	18.83	1,4A,7,7-tetramethyldecahydrocyclopropa[7,8]azuleno[3a,4-b]oxirene	C15H24O	220	0.85
21	19.07	Tibolone	C21H28O2	312	0.10
22	19.33	Acetic acid, 6-(1-hydroxymethyl-vinyl)-4,8a-dimethyl-3-oxo-1,2,3,5,6,7,8,8a-octahydronaphthalen-2-yl ester	C17H24O4	292	1.90
23	19.45	Gazaniolide	C15H18O2	230	0.18
24	20.48	Furosardonin A	C15H20O2	232	5.40
25	20.71	6á-Hydroxymethandienone	C20H28O3	316	0.18
28	21.58	4a-Methyl-1-methylene-1,2,3,4,4a,9,10,10a-octahydrophenanthrene	C16H20	212	6.13
30	22.12	Furosardonin A	C15H20O2	232	2.9
31	22.69	Pregn-4-EN-20-YN-3-one, 17-hydroxy-, (17à)-	C21H28O2	312	0.52
32	22.87	Ethanone, 2-(2,2,4,6,6-pentamethylcyclohexylidene)-1-phenyl-	C19H26O	270	1.5
33	23.18	8,11-Octadecadienoic acid, methyl ester	C19H34O2	294	0.61
34	23.28	10-Octadecenoic acid, methyl ester	C19H36O2	296	0.81
35	23.83	(3E,7E,11E)-1-Isopropyl-4,8,12-trimethylcyclotetradeca-3,7,11-trienol	C20H34O	290	0.68
36	24.01	Isopropyl-1,5,9-trimethyl-15-oxabicyclo[10.2.1]pentadeca-5,9-dien-2-ol	C20H34O2	306	0.71
37	25.81	1-Heptatriacotanol	C37H76O	536	1.16
38	26.50	Androstan-17-one, 3-ethyl-3-hydroxy-, (5à)-	C21H34O2	318	0.2
39	34.21	24-Norursa-3,12-diene	C29H46	394	0.31
40	34.74	Fucoxanthin	C42H58O6	658	1.41
41	35.46	24-Norursa-3,12-dien-11-one	C29H44O	408	0.11

**Table 3:** The primary chemical constituents found in chloroform extracts of *C. myrrha*.

No.	RT	Chemical Name (99.98%)	M. F	M. Wt	Area (%)
1	9.38	Cyclohexene, 4-ethenyl-4-methyl-3-(1-methylethenyl)-1-(1-methylethyl)-, (3R-trans)-	C15H24	204	1.01
2	10.14	.alfa.-Copaene	C15H24	204	0.69
5	10.85	1H-cycloprop[e]azulene, 1A,2,3,4,4A,5,6,7B-OCTAHYDRO-1,1,4,7-tetramethyl-, [1AR-(1Aà,4à,4Aá,7Bà)]-	C15H24	204	4.22
6	11.05	Caryophyllene	C15H24	204	9.48
7	11.42	ç-Elemene	C15H24	204	1.07
8	12.34	1,6-cyclodecadiene, 1-methyl-5-methylene-8-(1-methylethyl)-, [S-(E,E)]-	C15H24	204	1.32
9	12.45	Naphthalene, decahydro-4a-methyl-1-methylene-7-(1-methylethenyl)-, [4AR-(4Aà,7à,8Aá)]-	C15H24	204	1.55
10	13.35	Naphthalene, 1,2,3,5,6,8a-hexahydro-4,7-dimethyl-1-(1-methylethyl)-, (1S-CIS)-	C15H24	204	1.56
12	15.27	3,5,8a-trimethyl-4,4a,8a,9-tetrahydronaphtho[2,3-b]furan	C15H18O	214	2.66
13	16.13	Naphthalene, 4-methoxy-1,2,6,8-tetramethyl-	C15H18O	214	1.44
14	16.19	(4aS,8aS)-3,8a-Dimethyl-5-methylene-4,4a,5,6,8a,9-hexahydronaphtho[2,3-b]furan	C15H18O	214	32.41
15	16.29	5-isopropenyl-3,6-dimethyl-6-vinyl-4,5,6,7-tetrahydro-1-benzofuran	C15H20O	216	4.46
16	16.49	Cyclohexanemethanol, 4-ethenyl-à,à,4-trimethyl-3-(1-methylethenyl)-, acetate, [1R-(1à,3à,4á)]-	C17H28O2	264	0.57
17	16.85	(R,5E,9E)-8-Methoxy-3,6,10-trimethyl-4,7,8,11-tetrahydrocyclodeca[b]furan	C16H22O2	246	1.99
18	17.40	(R,5E,9E)-8-Methoxy-3,6,10-trimethyl-4,7,8,11-tetrahydrocyclodeca[b]furan	C16H22O2	246	6.84
19	17.70	7-tetracyclo[6.2.1.0(3.8)0(3.9)]undecanol, 4,4,11,11-tetramethyl-	C15H24O	220	10.77
20	18.24	Azulene, 1,2,3,4,5,6,7,8-octahydro-1,4-dimethyl-7-(1-methylethylidene)-, (1s-cis)-	C15H24	204	0.8
22	20.34	Furosardonin A	C15H20O2	232	2.08
23	20.88	Acetic acid, 6-(1-hydroxymethyl-vinyl)-4,8a-dimethyl-3-oxo-1,2,3,5,6,7,8,8a-octahydronaphthalen-2-yl ester	C17H24O4	292	4.89
24	21.49	4a-Methyl-1-methylene-1,2,3,4,4a,9,10,10a-octahydrophenanthrene	C16H20	212	1.00
25	21.63	Meso 1,1′-BI(5,5′-dimethoxy-2,2,2′,2′-tetramethylindan)	C24H30O2	350	1.62
26	21.98	4-(bicyclo[4.1.0]hept-7-ylidenemethyl)phenyl methyl ether	C15H18O	214	1.84
27	22.69	(5R,6S,8S,Z)-8-Methoxy-3,6,10-trimethyl-4-oxo-4,5,6,7,8,11-hexahydrocyclodeca[b]furan-5-yl acetate	C18H24O5	320	1.44
28	22.90	Morphina-2,4,6á-triol, N-formyl-	C17H21NO4	303	1.96
29	23.96	Isopropyl-1,5,9-trimethyl-15-oxabicyclo[10.2.1]pentadeca-5,9-dien-2-ol	C20H34O2	306	1.62

**Table 4:** Effects of *C. myrrha* extracts on larval mortality of *A. aegypti* and adult emergence percentage, 24 h post-treatment.

Plant Solvent	Conc. (ppm)	Mortality % (Mean ± SE)			Adult Emergence %
		Larval	Pupal	Larval–Pupal *	
Acetone	Control	0.00 ± 0.0 ^f^	1.67 ± 1.67 ^d^	1.67 ± 1.67 ^f^	98.33 ± 1.67 ^a^
	100	15.00 ± 2.89 ^e^	5.00 ± 1.61 ^bc^	20.00 ± 5.77 ^e^	80.00 ± 2.89 ^b^
	200	41.67 ± 3.33 ^d^	8.33 ± 1.69 ^b^	50.00 ± 2.89 ^d^	50.00 ± 5.77 ^c^
	300	61.67 ± 1.67 ^c^	13.33 ± 0.98 ^a^	75.00 ± 0.00 ^c^	25.00 ± 2.89 ^d^
	500	75.00 ± 2.89 ^b^	13.33 ± 1.33 ^a^	88.33 ± 1.67 ^b^	11.67 ± 1.67 ^e^
	1000	95.00 ± 2.89 ^a^	3.33 ± 0.87 ^cd^	98.33 ± 1.67 ^a^	1.67 ± 1.67 ^f^
Methanol	Control	0.00 ± 0.0 ^f^	3.33 ± 1.67 ^c^	3.33 ± 1.67 ^f^	96.67 ± 1.67 ^a^
	100	11.67 ± 1.67 ^e^	3.33 ± 1.91 ^c^	15.00 ± 2.89 ^e^	85.00 ± 5.77 ^b^
	200	35.00 ± 5.00 ^d^	8.33 ± 1.47 ^ab^	43.33 ± 4.41 ^d^	56.67 ± 3.33 ^c^
	300	56.67 ± 1.67 ^c^	11.66 ± 1.34 ^a^	68.33 ± 1.67 ^c^	31.67 ± 4.41 ^d^
	500	73.33 ± 1.67 ^b^	11.67 ± 1.01 ^a^	85.00 ± 2.89 ^b^	15.00 ± 2.89 ^e^
	1000	90.00 ± 2.89 ^a^	5.00 ± 0.40 ^bc^	95.00 ± 2.89 ^a^	5.00 ± 2.89 ^f^
Chloroform	Control	0.00 ± 0.0 ^f^	1.67 ± 1.67 ^c^	1.67 ± 1.67 ^e^	98.33 ± 1.67 ^a^
	100	16.67 ± 1.67 ^e^	8.33 ± 1.09 ^b^	25.00 ± 2.89 ^d^	75.00 ± 2.89 ^b^
	200	43.33 ± 4.41 ^d^	16.67 ± 1.29 ^a^	60.00 ± 5.77 ^c^	40.00 ± 2.89 ^c^
	300	65.00 ± 2.89 ^c^	18.33 ± 7.05 ^a^	83.33 ± 3.33 ^b^	16.67 ± 3.33 ^d^
	500	80.00 ± 5.00 ^b^	16.67 ± 4.22 ^a^	96.67 ± 6.01 ^a^	3.33 ± 1.67 ^e^
	1000	100.00 ± 0.0 ^a^	0.00 ± 0.0 ^c^	100.00 ± 0.0 ^a^	0.00 ± 0.0 ^f^

* Larval–pupal mortality refers to the total mortality rate of both larvae and pupae combined. ^a–f^: Different superscript letters within each treatment (column) express significant variation at a probability level of 0.0001. There is no significant difference at a probability level of >0.05 between any two means within the same column and with the same superscript letter.

**Table 5:** Larval mortality and lethal time values of *C. myrrha* resin extracts against *A. aegypti*, 24 h post-treatment.

Plant Extract	Conc. (ppm)	Mortality (%)	LC_50_ (Low.–Up.)	LC_90_ (Low.–Up.)	LC_95_ (Low.–Up.)	Slope	*X* ^2^
Acetone	Control	0.00 ± 0.0 ^f^	246.28 (218.67–275.15)	788.05 (663.69–984.91)	1095.83 (889.04–1445.83)	2.537 ± 0.204	1.135
	100	15.00 ± 2.89 ^e^					
	200	41.67 ± 3.33 ^d^					
	300	61.67 ± 1.67 ^c^					
	500	75.00 ± 2.89 ^b^					
	1000	95.00 ± 2.89 ^a^					
Methanol	Control	0.00 ± 0.0 ^f^	281.83 (250.40–316.23)	923.76 (760.43–1197.88)	1293.35 (1021.25–1782.63)	2.485 ± 0.212	1.109
	100	11.67 ± 1.67 ^e^					
	200	35.00 ± 5.00 ^d^					
	300	56.67 ± 1.67 ^c^					
	500	73.33 ± 1.67 ^b^					
	1000	90.00 ± 2.89 ^a^					
Chloroform	Control	0.00 ± 0.0 ^f^	224.36 (201.01–248.50)	608.39 (522.65–740.97)	807.21 (671.68–1030.38)	2.958 ± 0.242	4.744
	100	16.67 ± 2.89 ^e^					
	200	43.33 ± 7.64 ^d^					
	300	65.00 ± 5.00 ^c^					
	500	80.00 ± 8.66 ^b^					
	1000	100.00 ± 0.0 ^a^					

^a–f^: Different superscript letters within each treatment (column) express significant variation at a probability level of 0.0001. There is no significant difference at a probability level of >0.05 between any two means within the same column and with the same superscript letter.

**Table 6:** Larval mortality and lethal time values of *C. myrrha* resin extracts against *A. aegypti*, 48 h post-treatment.

Plant Extract	Conc. (ppm)	Mortality (%)	LC_50_ (Low.–Up.)	LC_90_ (Low.–Up.)	LC_95_ (Low.–Up.)	Slope	*X* ^2^
Acetone	Control	1.67 ± 1.67 ^f^	176.07 (156.05–195.70)	461.32 (400.57–554.22)	606.16 (510.35–763.33)	3.063 ± 0.265	1.545
	100	25.00 ± 2.89 ^e^					
	200	58.33 ± 7.26 ^d^					
	300	73.33 ± 4.41 ^c^					
	500	91.67 ± 4.41 ^b^					
	1000	100.00 ± 0.0 ^a^					
Methanol	Control	1.67 ± 1.67 ^f^	203.30 (179.88–226.93)	593.38 (505.82–731.56)	803.90 (661.42–1045.10)	2.754 ± 0.235	0.323
	100	20.00 ± 2.89 ^e^					
	200	51.67 ± 4.41 ^d^					
	300	68.33 ± 1.67 ^c^					
	500	86.67 ± 1.67 ^b^					
	1000	96.67 ± 3.33 ^a^					
Chloroform	Control	1.67 ± 2.89 ^f^	159.29 (141.15–176.72)	398.56 (353.83–461.62)	516.89 (447.85–624.30)	3.217 ± 0.253	0.670
	100	28.33 ± 5.77 ^e^					
	200	61.67 ± 10.4 ^d^					
	300	80.00 ± 5.00 ^c^					
	500	96.67 ± 2.89 ^b^					
	1000	100.00 ± 0.0 ^a^					

^a–f^: Different superscript letters within each treatment (column) express significant variation at a probability level of 0.0001. There is no significant difference at a probability level of >0.05 between any two means, within the same column and with the same superscript letter.

**Table 7:** Larval mortality and lethal time values of *C. myrrha* resin extracts against *A. aegypti*, 72 h post-treatment.

Plant Extract	Conc. (ppm)	Mortality (%)	LC_50_ (Low.–Up.)	LC_90_ (Low.–Up.)	LC_95_ (Low.–Up.)	Slope	*X* ^2^
Acetone	Control	3.33 ± 1.67 ^e^	127.67 (111.54–142.43)	291.03 (257.55–341.23)	367.60 (316.89–450.38)	3.581 ± 0.349	2.590
	100	38.33 ± 4.41 ^d^					
	200	76.67 ± 6.01 ^c^					
	300	88.33 ± 7.26 ^b^					
	500	100.0 ± 0.0 ^a^					
	1000	100.0 ± 0.0 ^a^					
Methanol	Control	3.33 ± 1.67 ^e^	142.13 (125.72–157.49)	323.33 (285.27–381.31)	408.16 (350.44–503.09)	3.590 ± 0.343	4.496
	100	33.33 ± 3.33 ^d^					
	200	70.00 ± 5.0 ^c^					
	300	85.00 ± 5.0 ^b^					
	500	100.0 ± 0.0 ^a^					
	1000	100.0 ± 0.0 ^a^					
Chloroform	Control	1.67 ± 2.89 ^e^	118.33 (103.58–131.64)	251.14 (224.64–289.64)	310.86 (271.90–372.67)	3.921 ± 0.384	0.817
	100	40.00 ± 5.0 ^d^					
	200	81.67 ± 5.77 ^c^					
	300	96.67 ± 5.77 ^b^					
	500	100.0 ± 0.0 ^a^					
	1000	100.0 ± 0.0 ^a^					

^a–e^: Different superscript letters within each treatment (column) express significant variation at a probability level of 0.0001. There is no significant difference at a probability level of >0.05 between any two means, within the same column and with the same superscript letter.

**Table 8:** The effects of *C. myrrha* plant extracts on oviposition, egg hatching rates, non-hatching eggs, and the fecundity of *A. aegypti*.

Plant Extract	Conc. (ppm)	No. of Eggs Laid	Hatching (%)	Fecundity (%)	Non-Hatching (%)	No. of Non-Hatched Eggs	
						Embryo (%)	Non-Embryo (%)
Acetone	Control	1412.00 ^a^	95.33 ^a^	100.00 ^a^	4.67 ^d^	1.35 ^c^	3.33 ^b^
	100	1372.00 ^a^	95.85 ^a^	97.17 ^ab^	4.15 ^d^	1.82 ^b^	4.45 ^b^
	200	1285.00 ^a^	91.60 ^ab^	91.01 ^b^	8.40 ^cd^	1.32 ^c^	7.08 ^b^
	300	1023.00 ^b^	82.99 ^ab^	72.45 ^c^	17.01 ^bcd^	1.96 ^b^	15.05 ^b^
	500	702.00 ^c^	74.07 ^b^	49.72 ^d^	25.93 ^bc^	1.85 ^b^	24.07 ^b^
	1000	382.00 ^d^	49.74 ^c^	27.05 ^e^	50.26 ^a^	2.62 ^a^	55.76 ^a^
Methanol	Control	1454.00 ^a^	97.94 ^a^	100.00 ^a^	2.06 ^b^	0.96 ^d^	1.10 ^b^
	100	1433.00 ^a^	97.00 ^a^	98.56 ^a^	3.00 ^b^	1.47 ^bc^	1.54 ^b^
	200	1386.00 ^ab^	93.22 ^a^	95.32 ^ab^	6.78 ^b^	1.37 ^bc^	5.34 ^ab^
	300	1240.00 ^b^	91.69 ^a^	85.28 ^b^	8.31 ^b^	1.29 ^cd^	7.02 ^ab^
	500	811.00 ^c^	86.19 ^ab^	55.78 ^c^	13.81 ^ab^	1.73 ^ab^	12.08 ^ab^
	1000	424.00 ^d^	69.58 ^b^	29.16 ^d^	30.42 ^a^	1.89 ^a^	28.54 ^a^
Chloroform	Control	1513.00 ^a^	97.22 ^a^	100.00 ^a^	2.78 ^c^	0.86 ^c^	1.92 ^c^
	100	1475.00 ^a^	95.80 ^a^	97.49 ^a^	4.20 ^c^	0.75 ^c^	5.49 ^c^
	200	1251.00 ^b^	89.21 ^a^	82.68 ^b^	10.79 ^c^	0.72 ^c^	10.07 ^c^
	300	1001.00 ^c^	83.12 ^a^	66.16 ^c^	16.88 ^bc^	0.80 ^c^	17.18 ^c^
	500	466.00 ^d^	68.03 ^b^	30.80 ^d^	31.97 ^b^	1.29 ^b^	45.71 ^b^
	1000	234.00 ^e^	12.82 ^c^	15.47 ^e^	87.18 ^a^	1.71 ^a^	98.29 ^a^

^a–e^: Different superscript letters within each treatment (column) express significant variation at a probability level of 0.0001. There is no significant difference at a probability level of >0.05 between any two means, within the same column and with the same superscript letter.

## Data Availability

Data are contained within the article.
